# Fragile X Mental Retardation Protein Regulates Activity-Dependent Membrane Trafficking and *Trans*-Synaptic Signaling Mediating Synaptic Remodeling

**DOI:** 10.3389/fnmol.2017.00440

**Published:** 2018-01-12

**Authors:** James C. Sears, Kendal Broadie

**Affiliations:** ^1^Department of Biological Sciences, Vanderbilt University, Nashville, TN, United States; ^2^Vanderbilt Kennedy Center for Research on Human Development, Nashville, TN, United States; ^3^Vanderbilt Brain Institute, Vanderbilt University Medical Center, Nashville, TN, United States

**Keywords:** fragile X syndrome, critical period, signaling, synapse, Drosophila

## Abstract

Fragile X syndrome (FXS) is the leading monogenic cause of autism and intellectual disability. The disease arises through loss of fragile X mental retardation protein (FMRP), which normally exhibits peak expression levels in early-use critical periods, and is required for activity-dependent synaptic remodeling during this transient developmental window. FMRP canonically binds mRNA to repress protein translation, with targets that regulate cytoskeleton dynamics, membrane trafficking, and *trans*-synaptic signaling. We focus here on recent advances emerging in these three areas from the *Drosophila* disease model. In the well-characterized central brain mushroom body (MB) olfactory learning/memory circuit, FMRP is required for activity-dependent synaptic remodeling of projection neurons innervating the MB calyx, with function tightly restricted to an early-use critical period. FMRP loss is phenocopied by conditional removal of FMRP only during this critical period, and rescued by FMRP conditional expression only during this critical period. Consistent with FXS hyperexcitation, FMRP loss defects are phenocopied by heightened sensory experience and targeted optogenetic hyperexcitation during this critical period. FMRP binds mRNA encoding *Drosophila* ESCRTIII core component Shrub (human CHMP4 homolog) to restrict Shrub translation in an activity-dependent mechanism only during this same critical period. Shrub mediates endosomal membrane trafficking, and perturbing Shrub expression is known to interfere with neuronal process pruning. Consistently, FMRP loss and Shrub overexpression targeted to projection neurons similarly causes endosomal membrane trafficking defects within synaptic boutons, and genetic reduction of Shrub strikingly rescues *Drosophila* FXS model defects. In parallel work on the well-characterized giant fiber (GF) circuit, FMRP limits iontophoretic dye loading into central interneurons, demonstrating an FMRP role controlling core neuronal properties through the activity-dependent repression of translation. In the well-characterized *Drosophila* neuromuscular junction (NMJ) model, developmental synaptogenesis and activity-dependent synaptic remodeling both require extracellular matrix metalloproteinase (MMP) enzymes interacting with the heparan sulfate proteoglycan (HSPG) glypican dally-like protein (Dlp) to restrict *trans*-synaptic Wnt signaling, with FXS synaptogenic defects alleviated by both MMP and HSPG reduction. This new mechanistic axis spanning from activity to FMRP to HSPG-dependent MMP regulation modulates activity-dependent synaptogenesis. We discuss future directions for these mechanisms, and intersecting research priorities for FMRP in glial and signaling interactions.

## Introduction

Nascent neural circuitry, while functional, is nevertheless still developing and initially manifests activity-dependent refinement and optimization. During early-use critical periods, new neural circuits are highly sensitive to sensory experience, exhibiting a transient window of heightened synaptic remodeling capacity (Hensch, 2004). Sensory input driving downstream circuit activity can result in persistent, long-lasting structural and functional changes, which generally cannot be retrained once the critical period has past (Takesian and Hensch, 2013). During this activity-dependent refinement, excitatory and inhibitory synapses are balanced in circuits, generally by removing excess excitatory synapses and adding new inhibitory synapses, thereby establishing an optimized excitatory/inhibitory (E/I) balance (Doll and Broadie, 2014). Therefore, suitably primed activity-dependent mechanisms must be present to sculpt synaptic connectivity during these critical periods. The fragile X mental retardation protein (FMRP), which when lost through epigenetic silencing of the *FMR1* gene results in fragile X syndrome (FXS), is a prime candidate for mediating activity-dependent synaptic remodeling during critical periods. FMRP is directly regulated by activity (Weiler et al., 1997; Antar et al., 2004) and, in turn, regulates activity-dependent processes (Huber et al., 2002; Li et al., 2002). Importantly, considerable evidence supports the theory that FXS is caused by excessive excitatory neurotransmission (hyperexcitation theory), reduced inhibitory transmission (hypoinhibition theory), or some combination of both, resulting in an excitation/inhibition imbalance (E/I imbalance theory) (Gibson et al., 2008; Cea-Del Rio and Huntsman, 2014).

The *Drosophila* FXS disease model has established conserved requirements for *Drosophila FMR1* (*dfmr1)* (Coffee et al., 2010, 2012). *Drosophila* FMRP has key roles in synaptic remodeling ranging from the larval neuromuscular junction (NMJ) and sensory circuits, to adult circadian clock neurons and the mushroom body (MB) olfactory learning/memory circuitry (Zhang et al., 2001; Pan et al., 2004; Gatto and Broadie, 2009; Gatto et al., 2014). Null *dfmr1* mutants display an elevated number of immature synaptic connections in these diverse circuits, as well as the loss of activity-dependent synaptic pruning (Gatto and Broadie, 2008; Tessier and Broadie, 2008). Importantly, *Drosophila* FMRP is developmentally regulated: FMRP levels are at their highest during very late pupal brain development and the first day of post-eclosion adulthood, with levels then decreasing dramatically at maturity (Tessier and Broadie, 2008). FMRP is required developmentally for synaptogenesis, bouton elimination/pruning, activity-dependent refinement and calcium signaling (Gatto and Broadie, 2008, 2009; Tessier and Broadie, 2008, 2011; Doll and Broadie, 2015, 2016). For E/I balance, *Drosophila* FMRP drives use-dependent down-regulation of synaptic excitability via metabotropic glutamate receptors (mGluRs) (Pan and Broadie, 2007; Pan et al., 2008; Repicky and Broadie, 2008), and promotes GAD levels and GABAergic innervation (Gatto et al., 2014). Given E/I balance is established during the critical period, FMRP loss during this developmental window consistently causes differential activity regulation of excitatory vs. inhibitory neurons in the *Drosophila* FXS model, with defective activity-dependent synapse morphogenesis and Ca^2+^ signaling maturation (Doll and Broadie, 2015, 2016).

Fragile X mental retardation protein is an RNA-binding translation repressor (Laggerbauer et al., 2001; Li et al., 2001; Darnell et al., 2011; Ascano et al., 2012; Chen and Joseph, 2015), with translation enhancement also reported (Todd et al., 2003; Muddashetty et al., 2007; Kenny et al., 2014; Fernandez et al., 2015; Kenny and Ceman, 2016). Primary established targets of repression include cytoskeletal and signaling regulators (Zhang et al., 2001, 2005; Lee et al., 2003; Bongmba et al., 2011; Santoro et al., 2012; Friedman et al., 2013; Majumder et al., 2016). Genetic and pharmacological correction of protein levels or elevated signaling in FXS models can rescue synaptic defects. For example, FMRP binds the mRNA encoding microtubule-associated protein IB (MAPIB)/Futsch, *dfmr1* null animals overexpress Futsch, and genetic Futsch reduction corrects synaptic structure/function defects in the *Drosophila* FXS model (Zhang et al., 2001). Likewise, FMRP binds the mRNA encoding actin-binding Profilin/Chickadee to suppress Chickadee levels, with Chickadee overexpression phenocopying *dfmr1* null defects, and decreasing Chickadee levels correcting synaptic defects (Reeve et al., 2005). In signaling, *Drosophila* FMRP genetically interacts with a mGluR in a bidirectional mechanism controlling ionotropic glutamate receptor (iGluR) classes to regulate synaptic function (Pan and Broadie, 2007; Repicky and Broadie, 2008). *Drosophila* FMRP also limits two heparan sulfate proteoglycan (HSPG) co-receptors that modulate *trans-*synaptic signaling, and genetic reduction of these HSPGs suppresses synaptic structure/function defects in the *Drosophila* FXS model (Friedman et al., 2013). Thus, FMRP targets regulating cytoskeletal and signaling dynamics are causally related to synaptic defects characterizing the FXS disease state. The discovery/ordering of such targets is critical for understanding the FXS disease state.

Mouse and *Drosophila* FXS models have been utilized to discover and test targets for therapeutic intervention. For example, inhibition of GSK3β/Shaggy with lithium has mediated promising effects (Klein and Melton, 1996; Stambolic et al., 1996; McBride et al., 2005; Choi et al., 2010; Mines and Jope, 2011). Therapeutic targets regulating the cytoskeleton have long been a focus of FXS model tests. For example, FMRP translationally represses Rac1, and Rac1 activity is elevated in FXS models (Lee et al., 2003; Bongmba et al., 2011; Majumder et al., 2016). Importantly, many Rac1 inhibitors are known which may hold therapeutic potential for FXS treatments (Tejada-Simon, 2015). Downstream of Rac1, inhibition of p21-activated kinase (PAK) signaling can prevent phenotypes in the mouse FXS disease model (Dolan et al., 2013). Classic work showed FXS patient-derived cells have reduced cAMP levels and induction (Berry-Kravis and Huttenlocher, 1992; Berry-Kravis et al., 1995). Mouse and *Drosophila* FXS models similarly show reduced cAMP levels, with Forskolin stimulation of cAMP production significantly diminished, and genetic/pharmacological correction of cAMP levels preventing FXS phenotypes (Kelley et al., 2007; Kanellopoulos et al., 2012). As a final example, the MMP-9 inhibitor minocycline has been shown in mouse and *Drosophila* FXS models to correct FXS phenotypes (Bilousova et al., 2008; Siller and Broadie, 2011). These strategies highlight mechanisms causally involved in FXS, with the recurrent theme of efficacious inhibition of targets hyper-activated in the disease state. Further investigation of these core pathways in FXS patients and models will likely lead to future clinically relevant discoveries.

Fragile X mental retardation protein plays key roles in the regulation of intercellular interactions governing synaptic remodeling, including *trans*-synaptic signaling and glial pruning. Work over the last several years has established that FMRP regulates *trans*-synaptic signaling at the *Drosophila* NMJ model synapse, particularly in the control of the founding Wnt Wingless (Wg) signaling pathway (Siller and Broadie, 2011; Friedman et al., 2013). Wg *trans*-synaptic signaling regulates activity-dependent synaptic structure/function remodeling (Ataman et al., 2008), with the Wg secreted from synapse-associated glia selectively regulating post-synaptic assembly and transmission strength (Kerr et al., 2014). Activity-dependent Wg signaling occurs in a very rapid time frame; for example, the Wg-driven formation of nascent presynaptic boutons (“ghost boutons”) occurs within minutes of stimulation (Ataman et al., 2008). Wg *trans*-synaptic signaling is modulated by extracellular HSPGs [e.g., dally-like protein (Dlp)] and matrix metalloprotease (MMP) enzymes that co-regulate each other in the synaptomatrix surrounding synaptic boutons (Dear et al., 2016). Importantly, HSPG/MMP levels and Wg signaling are altered in parallel in *dfmr1* null animals, and the genetic reduction of Dlp, or genetic/pharmacological reduction of secreted MMP1, both correct *Drosophila* FXS disease model phenotypes (Siller and Broadie, 2011; Friedman et al., 2013). In addition to the above glial involvement in *trans*-synaptic signaling, glia have also been implicated in neural phagocytosis pruning during remodeling (Tasdemir-Yilmaz and Freeman, 2014). Thus, glia may play central roles during FMRP-dependent synaptic refinement in response to activity states and intercellular signaling cues.

In this review, we focus on recent *Drosophila* FXS model studies of FMRP in activity-dependent synaptic remodeling. We highlight roles in a range of disparate neural circuits: (1) the adult central brain MB learning/memory circuit during an early-use critical period (Guven-Ozkan and Davis, 2014), (2) the adult giant fiber (GF) escape circuit connecting sensory input to motor output (Boerner and Godenschwege, 2010), and (3) the larval NMJ glutamatergic model synapse (Harris and Littleton, 2015). We concentrate on recent 2017 papers assaying different facets of FMRP biology in these circuits. In the MB circuit, FMRP functions in an activity sensor mechanism to mediate sensory experience refinement of olfactory projection neuron synapses during an early-use critical period, with loss of FMRP resulting in a hyper-excited state that is phenocopied in wildtype animals with intense stimulation (Doll et al., 2017). FMRP suppresses translation of ESCRTIII core component Shrub to enable endosomal membrane trafficking required for critical period activity-dependent synaptic refinement (Vita and Broadie, 2017). In the GF circuit, FMRP limits small molecule permeation in central interneurons, which is disrupted in the *Drosophila* FXS model (Kennedy and Broadie, 2017). At the NMJ, activity regulates extracellular HSPG/MMP co-localization in the synaptomatrix, within a FMRP-dependent mechanism driving synaptic remodeling (Dear et al., 2017). We end by discussing future directions stemming from this work, as well as emerging avenues on cAMP signal transduction, cytoskeleton regulation, glial-dependent refinement and activity-dependent *trans*-synaptic signaling impacting the FXS disease state.

## FMRP Requirements in Critical Period Activity-Dependent Synaptic Remodeling

**FIGURE 1:**
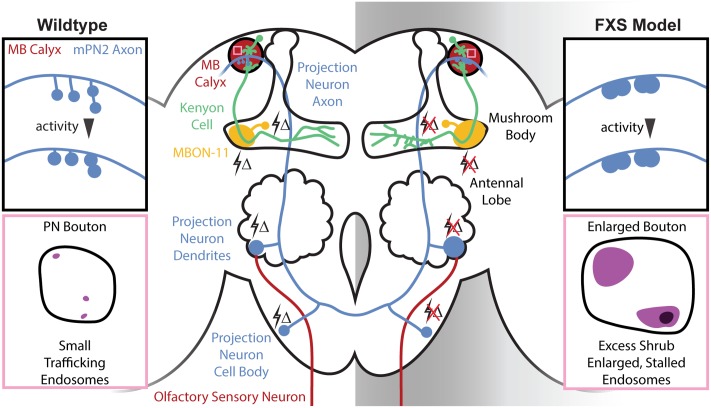
Central brain mushroom body (MB) circuit defects in the *Drosophila* Fragile X syndrome (FXS) model. Schematic of the *Drosophila* central brain olfactory circuitry comparing wildtype (Left) and the FXS disease model (Right). Olfactory sensory neurons (OSNs) (red, bottom) expressing specific odorant receptors converge in antennal lobe (AL) glomeruli to synapse on projection neurons (blue, middle). Projection neurons output to the MB calyx (red, top) to synapse on Kenyon cells (KCs) (green), which in turn project to MB axonal lobes to synapse on MB output neurons [e.g., MB output neuron type 11 (MBON-11, yellow)]. Changes in olfactory sensory experience (lightning bolts Δ) drive activity-dependent synaptic remodeling throughout this circuit in the early-use critical period, which fails in the FXS condition. Top insets (black boxes): schematic of MB calyx in wildtype and the FXS model. Projection neuron synaptic termini are normally subject to activity-dependent remodeling, but this is absent in the FXS model. The resulting collapsed synaptic architecture with enlarged boutons is phenocopied with strong activity in wildtype. Bottom insets (pink boxes): schematic of single projection neuron synaptic boutons in the wildtype and FXS model MB calyx. The endosomal sorting complex required for transport III (ESCRTIII) core component Shrub normally mediates rapid endocytic membrane trafficking within the PN synaptic boutons, but the FXS model displays an increased number of trafficking-arrested, enlarged synaptic endosomes.

The *Drosophila* MB olfactory learning and memory circuit in the developing adult brain has numerous advantages for researching critical periods. With a particularly well-defined neural circuitry map, coupled to a host of genetic tools and transgenic markers, we can probe the mechanisms of activity-dependent remodeling in individually identified single neurons (**Figure 1**). Olfactory sensory experience can be manipulated in developmental time periods, or different neurons within the defined circuit targeted with bidirectional optogenetics or transgenic toxins, to dissect activity-dependent remodeling in this rapidly developing animal model. In this defined neural circuit, olfactory sensory neurons (OSNs) expressing the same odorant receptor converge on fully mapped antennal lobe (AL) synaptic glomeruli to innervate central brain projection neurons (PNs; **Figure 1**). PNs output information to the MB calyx by synapsing on Kenyon cells (KCs) involved in learning acquisition and memory consolidation (**Figure 1**). Using KC clonal analyses, we first discovered that FMRP is required for activity-dependent synaptic pruning downstream of olfactory sensory experience, and in response to targeted optogenetic depolarization (Tessier and Broadie, 2008). Sensory experience and activity both promote FMRP expression, with FMRP levels elevated during late pupariation and the first day post-eclosion (1 dpe), but much lower at maturity (e.g., 7 dpe). During this transient window, FMRP represses overall protein levels as well as specific FMRP targets (e.g., Profilin/Chickadee; Tessier and Broadie, 2008). This work established an FMRP-defined critical period in the MB circuit for early-use, activity-dependent circuit refinement.

The recent emergence of new transgenic driver libraries allows for an unprecedented, circuit-level investigation of FMRP requirements during this critical period development (Jenett et al., 2012). These new generation, highly selective drivers allow neuron-specific visualization and optogenetic manipulation [e.g., excitatory olfactory PN type 2 (mPN2) and inhibitory MB output neuron type 11 (MBON-11); Aso et al., 2014; Ito et al., 2014]. Using these tools, the initial goal was to characterize activity-dependent synaptic remodeling during critical period development, and to test for FMRP requirements in this mechanism. In line with the excitatory vs. inhibitory neuron class (**Figure 1**), targeted optogenetic depolarization results in decreased dendritic size in mPN2 and opposite increase in dendritic arborization in MBON-11 neurons (Doll and Broadie, 2015). Consistently, prevention of depolarization through optogenetic hyperpolarization results in increased mPN2 dendritic arbors and a decrease in MBON-11 dendritic size. FMRP loss results in increased dendritic arborization in both neuron classes, and prevents activity-dependent remodeling due to either hyper- or hypo-polarization (Doll and Broadie, 2015). Crucially, these activity-dependent changes normally only occur during the early-use critical period (0–1 dpe), and FMRP is necessary only during this window for synaptic remodeling (Doll and Broadie, 2015). Therefore, neurons without FMRP cannot respond to activity, eliminating their capacity to be refined during circuit optimization (**Figure 1**). The wider implication of this insensitivity is that FXS disease state neurons are no longer able to mature based on critical period experience in order to fine-tune behavioral responses.

Most critical period activity-dependent refinement studies in this FXS model have been restricted to structural analyses. The one exception is testing the maturation of calcium signaling dynamics with transgenic GCaMP reporters (Doll and Broadie, 2016). In the same excitatory input mPN2 and inhibitory output MBON-11 neuronal pair (**Figure 1**), *dfmr1* null mPN2 shows strongly elevated depolarization-induced Ca^2+^ transients, whereas MBON-11 manifests an opposite Ca^2+^ signaling depression during the critical period (Doll and Broadie, 2016). As above with architecture, these functional phenotypes are restricted to the 0–1 dpe critical period window, with activity-dependent Ca^2+^ transients largely normalized to wildtype levels in both neuron classes by maturity (e.g., 7 dpe). Excitatory mPN2s manifest a persistent functional defect, with depolarization-induced Ca^2+^ transients shifted from elevated in the critical period to slightly depressed at maturity (Doll and Broadie, 2016). Importantly, cell-specific rescue of FMRP in the critical period restores Ca^2+^ signaling in both neuron classes, while conditional RNAi knockdown of FMRP phenocopies the *dfmr1* null defects, proving a cell-autonomous, critical period role for FMRP in Ca^2+^ signaling control (Doll and Broadie, 2016). In wildtype animals, targeted optogenetic depolarization during the critical period entrains increased Ca^2+^ transients in both neuron classes, but this activity-dependent plasticity is lost in the FXS model, with a slight timing delay in *dfmr1* null MBON-11 neurons (Doll and Broadie, 2016). These results suggest an E/I imbalance mechanism: excitatory neurons do not mature due to hyper-excitability, while inhibitory neurons do not mature due to hypo-excitability.

Most recently, mPN2 connectivity in the MB calyx learning/memory center was tested for FMRP requirements in activity-dependent remodeling (Doll et al., 2017). In these well-defined synapses, mPN2 axons project collateral branches with boutons into synaptic microglomeruli innervating KC dendrites (**Figure 1**). FMRP regulates mPN2-KC connectivity specifically during the critical period, with branch length decreased and synaptic bouton area increased in *dfmr1* nulls (Doll et al., 2017), causing a much more compact innervation pattern (**Figure 1**). In the mutants, mPN2 microglomeruli display a loss of the presynaptic active zone scaffold Bruchpilot during the critical period, suggesting delayed synaptogenesis (Doll et al., 2017). All defects are completely restricted to the critical period, with normal synaptic architecture and molecular differentiation restored by maturity (e.g., 7 dpe). GFP reconstitution across synaptic partners (GRASP) to test mPN2-KC connections (Feinberg et al., 2008; Pech et al., 2013) reveals that *dfmr1* null synaptic contacts are fewer in number, larger in size and more spatially restricted in the critical period, but not at maturity (**Figure 1**; Doll et al., 2017). EM ultrastructural analysis confirms that synaptic bouton size is increased in *dfmr1* null mutants during the critical period. Moreover, directly visualized T-bar synaptic active zones are drastically reduced in density in the FXS model, consistent with the loss of Bruchpilot labeling during the critical period (Doll et al., 2017). Given the activity-dependent remodeling during the normal critical period, and the activity insensitivity of *dfmr1* mutants, it was hypothesized that connectivity defects arise from activity-dependent refinement that occurs only in wildtype animals.

Odor response mapping studies demonstrate that IR75d OSNs respond to pyrrolidine upstream of mPN2 (**Figure 1**; Silbering et al., 2011; Münch and Galizia, 2016). Pyrrolidine exposure in the critical period, but not at maturity, phenocopies *dfmr1* synaptic defects and no changes occur in *dfmr1* mutants, demonstrating that FMRP is required for sensory experience synaptic remodeling (Doll et al., 2017). At maturity, pyrrolidine exposure causes no changes in wildtype animals, but does cause a reduction in *dfmr1* branch length, consistent with a shifted critical period. Optogenetic stimulation during the critical period also results in mPN2-KC connectivity changes in controls, but not *dfmr1* mutants (Doll et al., 2017). Conversely, targeted optogenetic hyperpolarization or tetanus toxin neurotransmission blockade both result in the opposite consequence of expanded MB calyx innervation in controls, but not *dfmr1* mutants (**Figure 1**). All manipulations show FMRP is required for activity-dependent synaptic remodeling in the critical period. One exception is hyperpolarization causes partial rescue of *dfmr1* bouton area, which may indicate an inhibitory mechanism that can still promote some synaptic refinement despite FMRP loss and decreased GABAergic function in the FXS model (Gatto et al., 2014). Indeed, GABA agonists can rescue hyperexcitation in FXS models (Chang et al., 2008; Olmos-Serrano et al., 2010), and activating inhibitory neurons can rescue the experience-driven remodeling (Fagiolini and Hensch, 2000; Hensch, 2004). This may provide a parallel to FMRP critical period requirements, where a weakened inhibitory influence might suppress critical period hyperexcitation in the FXS disease state.

Taken together, these new studies show a transient requirement for FMRP during the early-use sensory experience critical period of synaptic remodeling (Doll et al., 2017). The *Drosophila* FXS disease model presents synaptic connectivity characteristics replicated by strong developmental activation of the brain circuitry. We conclude, therefore, that FXS is a hyper-activated state, or responsive as if hyper-activated, and that FMRP normally functions in an activity-dependent mechanism to enable circuit refinement during the critical period (Doll et al., 2017). Given the developmental and activity-dependent regulation of FMRP, coupled to its maintained requirement in learning and memory, it is tempting to speculate that loss of FMRP only during this transient window results in persistent network defects at multiple levels, including hyperactivity and improper connectivity (Pan et al., 2004; McBride et al., 2005; Bolduc et al., 2008; Tessier and Broadie, 2008; Doll and Broadie, 2015, 2016; Doll et al., 2017). Moreover, the appearance of a shifted critical period (Doll et al., 2017) is consistent with the argument that delays and developmental perturbations during neural circuit and E/I refinement may result in persistent behavioral abnormalities (Harlow et al., 2010; Takesian and Hensch, 2013). While our current metrics indicate rectification of structural and functional defects following the critical period (Bureau et al., 2008; Doll et al., 2017), there is also apparent overcorrection and blunted calcium signaling at maturity (Tessier and Broadie, 2008; Doll and Broadie, 2016). Future work needs to dissect both transient critical period and lasting mature consequences of FMRP loss in the FXS brain circuitry.

## FMRP Role in Endocytic Membrane Trafficking during Synaptic Refinement

Fragile X mental retardation protein acts primarily as an mRNA-binding translation suppressor, so this function was explored to test mechanisms of activity-dependent critical period synaptic remodeling (Vita and Broadie, 2017). A *Drosophila* brain developmental proteomics screen was done to identify candidate protein changes occurring during the critical period window (Tessier and Broadie, 2012). A secondary screen tested for activity-regulated proteins, consistent with a role in developmental plasticity. Finally, candidate hit overexpression was assayed for predicted phenocopy of FXS defects, and protein level correction tested for predicted rescue of *dfmr1* null phenotypes during critical period development. A new FMRP target meeting all requirements is endosomal sorting complex required for transport III (ESCRTIII) core member Shrub (Vita and Broadie, 2017), *Drosophila* homolog of yeast Snf7/Vsp32 and human CHMP4 (Babst et al., 2002). Shrub exists as auto-inhibited monomers in the cytosol, which assemble in linear polymer arrays of spiral/helical filaments on membranes to drive inverse membrane budding (Teis et al., 2008). ESCRTIII mediates both plasma membrane and organelle trafficking (e.g., endosome-to-multivesicular body; MVB) in cooperation with other ESCRTs and the AAA-ATPase Vps4 (Henne et al., 2013). Canonically, ESCRTIII sorts ubiquitinated proteins to the lysosomal degradation pathway to remove targeted cell surface receptors (Sorkin, 1998; Babst et al., 2002). Importantly, ESCRTIII components are carefully regulated in endosome to MVB maturation, with loss or gain of ESCRTIII components resulting in similar trafficking aberrations, often in the form of greatly enlarged endosomal organelles (Teis et al., 2008).

**FIGURE 2:**
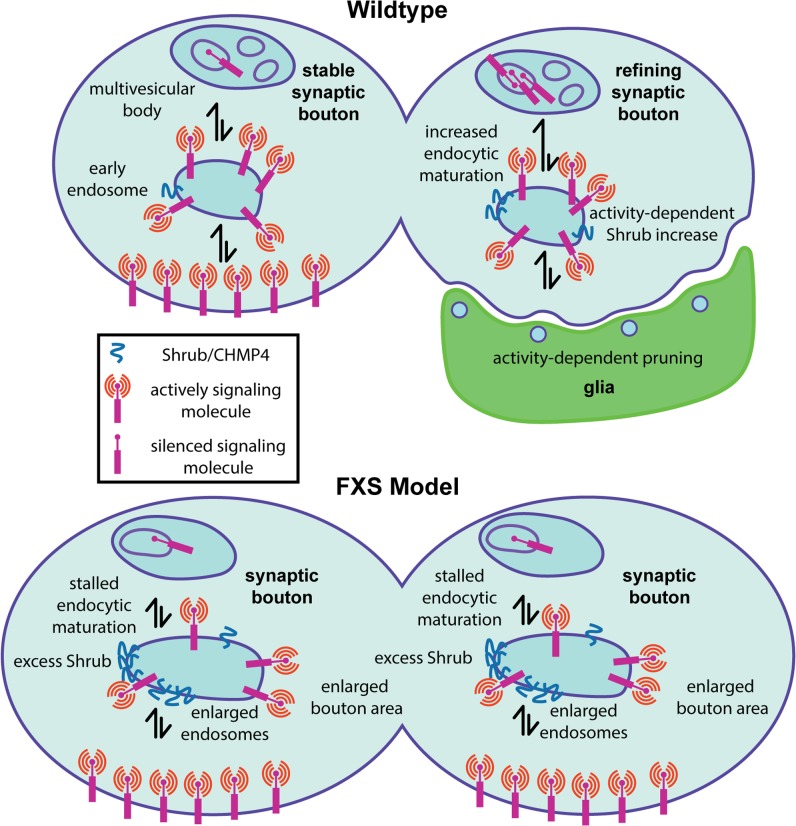
Presynaptic endosomal membrane trafficking defects in the *Drosophila* FXS model. Diagram summarizing a new fragile X mental retardation protein (FMRP) role in the regulation of presynaptic membrane trafficking by the ESCRTIII core component Shrub/CHMP4. **(Top)** In wildtype animals, appropriate Shrub levels mediate endosomal membrane trafficking within presynaptic boutons, which is required for activity-dependent synaptic pruning/refinement. It is hypothesized that activity-dependent endosomal trafficking regulates the presentation of surface signaling molecules that trigger phagocytosis by glia (green) during the early-use critical period. **(Bottom)** In the FXS disease model, excess Shrub translation leads to stalled endosomal membrane trafficking defects, resulting in enlarged endosomes within presynaptic boutons. It is hypothesized that impaired membrane signaling regulation via inappropriate presentation of surface cues driving glial phagocytosis prevents appropriate activity-dependent synaptic pruning/refinement.

In *Drosophila*, Shrub is necessary for developmental axonal pruning, as well as for limiting dendritic arborization (Sweeney et al., 2006). These precedents support a role for Shrub downstream of FMRP translational suppression in synaptic remodeling. A key distinction is that Shrub levels are elevated in the FXS model (Vita and Broadie, 2017), predicting defects caused by excess Shrub. Consistent with selective involvement in the critical period, Shrub levels are elevated in *dfmr1* null brains during the 0–1 dpe window defined above, and FMRP expression rescues Shrub levels during this period (Vita and Broadie, 2017). Importantly, optogenetic stimulation drives increased Shrub levels in wildtype animals during the critical period, whereas *dfmr1* mutants display no Shrub protein level changes, indicating FMRP mediates activity-dependent regulation. Employing RNA immunoprecipitation, it was found that FMRP binds *shrub* mRNA (Vita and Broadie, 2017). Taken together, these results demonstrate FMRP limits Shrub levels during the critical period by repressing translation in an activity-dependent mechanism (Vita and Broadie, 2017). With a restricted PN driver (Nrv3-Gal4) for projection neurons innervating the MB calyx (**Figure 1**), it was shown that Shrub overexpression and FMRP loss similarly cause overelaborated synaptic contacts during the critical period (Vita and Broadie, 2017). Moreover, EM ultrastructural analyses revealed Shrub overexpression and FMRP loss both result in enlarged PN synaptic boutons within the MB calyx (**Figures 1**, **2**). These results confirmed the importance of Shrub elevation in FXS phenotypes, and suggested that endocytic membrane trafficking is required for critical period synaptic refinement.

As a first step in assaying membrane trafficking, the endosome marker Rab5 was assayed in PN synaptic boutons innervating the MB calyx (Vita and Broadie, 2017). Both Shrub overexpression and FMRP loss result in an elevated number of enlarged Rab5-positive endosomes in PN synaptic boutons during the critical period (**Figures 1**, **2**). Consistently, ultrastructural analyses reveal strikingly enlarged endosomic vacuoles within PN synaptic boutons in both the Shrub overexpressing and *dfmr1* null animals (Vita and Broadie, 2017). Interestingly, both conditions also display an increased number of enlarged endosomal intraluminal vesicles, consistent with reports of Sfn7 overexpression and interpreted as a consequence of stalled MVB sorting (Teis et al., 2008). Taken together, these results suggest gain of Shrub or loss of FMRP similarly causes trafficking-arrested synaptic endosomes (**Figures 1**, **2**). To definitively test the FMRP/Shrub interaction in the context of the FXS disease model, Shrub levels were corrected (*shrub*/+ heterozygotes) in an otherwise *dfmr1* null mutant (Vita and Broadie, 2017). This correction rescues *dfmr1* phenotypes, with a significant restoration of PN innervation and synaptic bouton area, and complete rescue of endosome trafficking (**Figure 2**). This work establishes Shrub as an activity-dependent synaptic refinement protein, negatively regulated by FMRP during the critical period to mediate appropriate early-use neural circuit remodeling (Vita and Broadie, 2017). The mechanism likely involves Shrub-dependent endocytic trafficking, either of membrane being internalized during synaptic pruning, or in control of surface guidance molecules regulating activity-dependent synapse elimination (**Figure 2**).

It is tempting to speculate that stalled MVB maturation is a crucial determinant of the arrested critical period synaptic refinement characterizing the FXS disease state, operating via short-term plasma membrane and/or long-term signaling misregulation (Vita and Broadie, 2017). Evidence for the latter hypothesis comes from developmental pruning studies showing that reduction of cell adhesion molecule Neuroglian coincides with ESCRT-mediated pruning of sensory neuron dendrites during metamorphosis (Zhang et al., 2014). However, Neuroglian levels have not yet been demonstrated to be changed in the *Drosophila* FXS model, and Neuroglian is not known to be involved in MB synaptic pruning (Reeve et al., 2005; Zhang et al., 2014). Given Shrub is elevated with neuronal activity, we hypothesize it acts to sort activity-dependent reduction of as yet unidentified surface receptors regulating synaptic refinement (Vita and Broadie, 2017). One intriguing possibility is that Shrub-mediated membrane trafficking regulates cell surface signals for glial phagocytosis driving synaptic pruning during the early-use critical period (**Figure 2**). Consistently, *dfmr1* null mutants display delayed developmental MB gamma neuron pruning mediated by phagocytic glia and delayed glial engulfment of damaged axons, as well as clear deficiencies in immune cell-mediated engulfment (Tasdemir-Yilmaz and Freeman, 2014; O’Connor et al., 2017). Therefore, we hypothesize that activity-dependent defects in synaptic refinement in the FXS disease state could be due to improper intercellular interactions between neurons and glia (**Figure 2**), which depend on FMRP regulation of Shrub-mediated membrane trafficking.

Shrub misregulation is not the only aberrant translational repression in FXS, and there are broad consequences to neuron properties. Indeed, new evidence suggests this defect alters how molecules permeate *dfmr1* null neurons (Kennedy and Broadie, 2017). Iontophoresis of small polar dyes (e.g., neurobiotin, lucifer yellow) has long been used to assay gap junctions linking electrically coupled neurons (Lapper and Bolam, 1991; Hanani, 2012; Kudumala et al., 2013; Lee and Godenschwege, 2015), whereas large dyes (e.g., dextran-tetramethylrhodamine) fill single neurons without transfer (Phelan et al., 1996). The electrically coupled *Drosophila* giant fiber interneuron (GFI) transmitting information from sensory neuron inputs to motor neuron outputs (Allen et al., 1998) has been used extensively for such dye injection studies (Boerner and Godenschwege, 2010). Null *dfmr1* mutants have strong defects in GFI-dependent behaviors (Martinez et al., 2007), and was therefore targeted for studies of electrical and chemical synaptic connectivity in our FXS model. However, a surprising discovery was made; mutant GFI axons, dendrites and cell bodies are much more easily dye-loaded (Kennedy and Broadie, 2017). The striking defect is specific to small polar dyes, but cannot be attributed to altered electrical synapse coupling. FMRP is absolutely required, since neuron-targeted FMRP fully rescues defects. Membrane properties do not account for the difference, which is due to a highly elevated rate of cytosolic dye incorporation (Kennedy and Broadie, 2017). Our working hypothesis is that elevated protein levels caused by loss of FMRP translational suppression fundamentally alters the cytosolic milieu, to change molecular diffusion rates in FXS model neurons.

## FMRP Requirement in Activity-Dependent Proteolytic Synapse Remodeling

**FIGURE 3:**
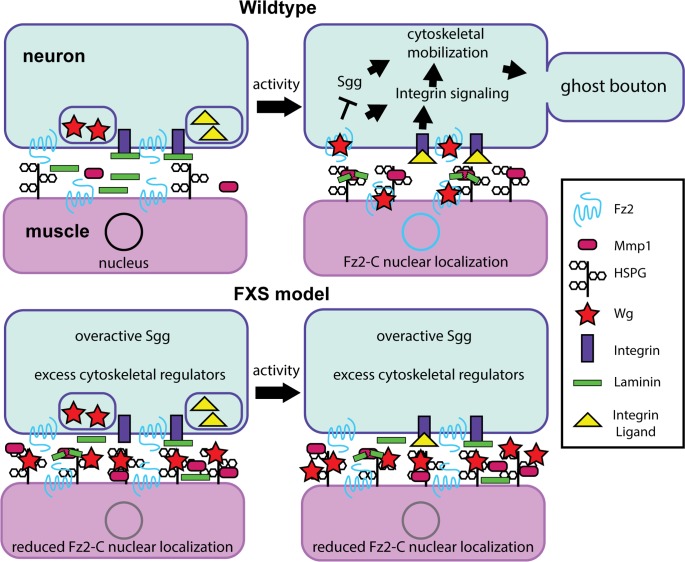
Synaptomatrix *trans*-synaptic signaling defects in the *Drosophila* FXS model. Diagram summarizing a new requirement for the secreted matrix metalloproteinase 1 (MMP1) during activity-dependent synaptic remodeling. **(Top)** In wildtype animals, an activity-dependent FMRP mechanism is required for neural activity to drive heparan sulfate proteoglycan (HSPG) dally-like protein (Dlp) localization at the synapse to recruit MMP1, whose enzymatic function is required for activity-dependent ghost bouton formation. HSPG-MMP1 directed proteolysis drives *trans*-synaptic Wnt Wingless (Wg) signaling for activity-dependent ghost bouton formation. Activity drives presynaptic signaling via the Frizzled-2 (Fz2) Wg receptor inhibiting GSK3β/Shaggy and integrin receptor signaling to control cytoskeleton dynamics, and post-synaptic Fz2 C-terminal cleavage and subsequent Fz2-C nuclear localization regulating new protein synthesis. It is hypothesized that MMP1 may cleave synaptomatrix Laminin to regulate ligand interactions with integrin receptors. **(Bottom)** In the FXS disease model, without FMRP Dlp and MMP1 are significantly increased at the synapse under basal resting conditions, and their levels do not change with activity manipulations. This activity-insensitivity prevents appropriate activity-dependent regulation of *trans*-synaptic signaling in the synaptomatrix, likely through inappropriate sequestration of the Wg ligand by HSPG Dlp. It is hypothesized that this defect is also linked to improper integrin signaling regulation.

Up to this point, we have focused on cell-autonomous FMRP requirements, yet a crucial aspect of synaptogenesis and synaptic refinement is coordinated, *trans*-synaptic signaling between partners (Barros et al., 2011; Dani and Broadie, 2012). This highly dynamic intercellular communication influences innervation patterns, synaptic architecture and neurotransmission strength, although roles in activity-dependent mechanisms are less clear (Barros et al., 2011; Dani and Broadie, 2012). The extensive toolkit available for the *Drosophila* glutamatergic NMJ model synapse is ideally suited for testing *trans*-synaptic signaling within activity-dependent mechanisms (Broadie et al., 2011; Harris and Littleton, 2015). Enlarged presynaptic boutons at the NMJ are easily distinguishable from the post-synaptic subsynaptic reticulum (SSR), and numerous genetic tools, markers and assays separate pre- versus post-synaptic requirements (Harris and Littleton, 2015). Signaling ligands must necessarily traverse the extracellular synaptomatrix. Two key synaptomatrix regulatory factors are (1) HSPGs and (2) matrix metalloproteinases (MMPs; **Figure 3**). HSPGs contain a core protein and heparan sulfate (HS) glycosaminoglycan (GAG) chains, which bind MMPs and extracellular signaling ligands (Park et al., 2000; Tocchi and Parks, 2013). HSPGs also link MMPs to their targets, promoting proteolytic activation/specificity (Tocchi and Parks, 2013). At the *Drosophila* NMJ, HSPGs regulate synaptic architecture, presynaptic active zone size/number and post-synaptic function, and serve to localize Wnt signaling ligands (Johnson et al., 2006; Dani et al., 2012; Kamimura et al., 2013).

In both mammalian synapses and the *Drosophila* NMJ model, extracellular MMPs directly and indirectly regulate the *trans*-synaptic signaling ligands modulating synaptic structure and function (Wlodarczyk et al., 2011; Dear et al., 2016). The mammalian genome encodes at least 24 MMPs with reportedly redundant/overlapping functions, many of which are localized to synapses. In contrast, *Drosophila* MMPs are represented by just two genes, *mmp1* and *mmp2*, which encode a single secreted and single GPI-anchored enzyme, respectively; although an anchored MMP1 has recently been described (Llano et al., 2000, 2002; LaFever et al., 2017). Compared to the MMP complexity in mammals, *Drosophila* enables reductionist testing of MMP roles in the FXS state. In *Drosophila*, both MMP1 and MMP2 regulate axonal and dendritic architecture (Kuo et al., 2005; Yasunaga et al., 2010; Depetris-Chauvin et al., 2014). At the *Drosophila* NMJ, both MMPs limit presynaptic growth, functional differentiation, and Wnt Wg *trans*-synaptic signaling (Dear et al., 2016). Interestingly, while MMP1 promotes MMP2 and HSPG Dlp localization, MMP2 limits MMP1 and Dlp localization at the synapse (Dear et al., 2016). These interactions suggest a complex level of interplay between MMPs and HSPGs within the synaptomatrix interface. Functionally, MMPs cleave not only extracellular matrix (ECM) targets during axon pathfinding (Miller et al., 2007, 2011), but also cell adhesion molecules (CAMs) in activity-dependent mechanisms sculpting synapse structure, turning off signaling and mobilizing membrane turnover in processes associated with neurological disorders including FXS (Nagappan-Chettiar et al., 2017).

In Wnt Wg signaling, presynaptic activity leads to Wg secretion, which binds to Frizzled-2 (Fz2) receptors on both pre- and post-synaptic cells (Koles and Budnik, 2012). Wg signaling drives both divergent and non-canonical Wnt cascades in pre- and post-synaptic cells (**Figure 3**), modulating both synapse structure and function (Koles and Budnik, 2012). Importantly, activity-induced Wg secretion drives activity-dependent synaptic remodeling, which operates within a rapid time-frame to promote formation of “ghost boutons”; immature boutons with presynaptic but not post-synaptic specialization (Ataman et al., 2008). Critically, extracellular HSPGs are integrally involved in Wg *trans*-synaptic signaling (**Figure 3**), highlighting the importance of the synaptomatrix in Wg signaling regulation (Harris and Littleton, 2015). Specifically, the secreted HSPG Perlecan balances pre- and post-synaptic Wg signaling by promoting post-synaptic Wg localization (Kamimura et al., 2013). Moreover, the GPI-anchored HSPG Dlp regulates Wg signaling in a concentration-dependent manner (**Figure 3**): based on Dlp co-receptor levels relative to Fz2 receptor and Wg ligand, Dlp can either restrict or promote Wg signaling as a negative and positive signaling regulator (Yan et al., 2009). Importantly, FMRP restricts synaptic levels of two HSPGs (Dlp and Syndecan) to regulate Wnt Wg *trans*-synaptic signaling, which is strongly misregulated in the FXS disease model (Friedman et al., 2013). Given the complex interactions between neural activity states, MMP proteolytic function, HSPG coreceptors and signaling mechanisms, activity-dependent Dlp-MMP interactions badly needed to be compared in normal versus FXS model synapses (Dear et al., 2017).

To test activity-dependent mechanisms, temperature-sensitive dTRPA1 channels were used to acutely depolarize neurons over a 1-h period (Hamada et al., 2008; Pulver et al., 2009). These studies demonstrated that MMP1, but not MMP2, is required to form ghost boutons (Dear et al., 2017). Consistently, dTRPA1 activation, or high [K^+^] depolarization for just 10 min, rapidly increases MMP1 at the synapse (**Figure 3**). Conversely, MMP2 is reduced by stimulation, as predicted since MMP1 limits MMP2 (Dear et al., 2016). Moreover, stimulated synapses rapidly elevate Dlp, with increased Dlp and MMP1 co-localization (Dear et al., 2017), supporting previous findings of genetic interaction at the NMJ. Importantly, the Dlp-Mmp1 co-localization in synaptic subdomains is significantly increased following acutely elevated neuronal activity in just 10 min (**Figure 3**). Since HSPGs are known to anchor proteases in other contexts (Tocchi and Parks, 2013), the dependence of MMP1 localization on Dlp was next tested. Both genetic mutant and targeted RNAi reduction of Dlp reduce synaptic MMP1 levels dramatically, whereas Dlp overexpression causes an opposing MMP1 increase at the synapse (Dear et al., 2017). These results show that the GPI-anchored Dlp regulates secreted MMP1 localization (**Figure 3**). Moreover, overexpression of Dlp lacking HS-GAG chains causes no change in MMP1 localization, suggesting that the HS-GAG chains are necessary for MMP1 synaptic localization (Dear et al., 2017). These results are consistent with other studies that have established roles for HS-GAG chains in HSPG activity at the synapse (Baeg et al., 2001; Johnson et al., 2006; Yan et al., 2009).

Given activity recruits HSPG Dlp, which in turn localizes MMP1 at the synapse, there is expected to be an activity-dependent increase in proteolytic activity surrounding synaptic boutons. To test this prediction, a dye-quenched fluorogenic gelatin substrate was tested in *in situ* zymography studies of protease enzymatic function (Siller and Broadie, 2011; Shilts and Broadie, 2017). Importantly, Dlp reduction results in reduced MMP-dependent proteolytic activity, while Dlp overexpression, with or without HS-GAG chains, elevates enzymatic function (Dear et al., 2017). These results are consistent with the hypothesis that synaptic Dlp levels tune synaptomatrix MMP1 proteolytic cleavage capacity, possibly via a Dlp core protein interaction resulting in protease activation (**Figure 3**). With the knowledge that basal MMP1 levels are tuned by membrane-anchored Dlp, it remained to be tested whether the acute neuronal activity-induced MMP1 increase also depends on Dlp. Indeed, Dlp loss suppresses activity-dependent MMP1 localization at the synapse, whereas Dlp overexpression elevates MMP1 levels and co-localization with Dlp (Dear et al., 2017). In line with above studies, overexpression of a Dlp isoform lacking HS-GAG chains results in a reduced activity-dependent enrichment of MMP1 at the synapse (**Figure 3**). Importantly, whereas synaptic MMP1 abundance tightly depends on Dlp, loss of MMP1 does not prevent the activity-dependent synaptic enrichment of Dlp (Dear et al., 2017). Thus, synaptic MMP1 localization depends on Dlp, but not vice versa. Taken together, these results support the conclusion that Dlp is absolutely necessary for the rapid activity-dependent synaptic localization of MMP1.

In the *Drosophila* FXS model, Dlp is constitutively elevated at the NMJ synapse, and reduction of Dlp (or dependent MMP1) in otherwise *dfmr1* null mutants suppresses FXS synaptogenic phenotypes (Siller and Broadie, 2011; Friedman et al., 2013). Therefore, activity-dependent Dlp and MMP1 synaptic enrichment was tested in the FXS model (Dear et al., 2017). As expected, MMP1 synaptic localization is strikingly increased in *dfmr1* null mutants (**Figure 3**). One interpretation is that this enrichment reflects a FXS hyper-excited state, manifested in elevated Dlp localization at the synapse (Friedman et al., 2013). Consistent with this idea, genetic reduction of Dlp restores normal MMP1 levels in *dfmr1* null synapses (Dear et al., 2017). Importantly, acute stimulation in *dfmr1* mutants causes no activity-dependent enrichment of MMP1 (Dear et al., 2017), demonstrating that MMP1 is insensitive to activity-dependent regulation in the FXS condition (**Figure 3**). Moreover, Dlp reduction restores activity-induced MMP1 enrichment in *dfmr1* null synapses (Dear et al., 2017). Just like stimulated controls, stimulated *dfmr1* nulls heterozygous for Dlp display striking synaptic enrichment of Mmp1 (**Figure 3**). Therefore, Dlp is the critical link determining activity-regulated synaptic MMP1 localization downstream of FMRP. These findings suggest MMP inhibition may ameliorate FXS phenotypes; for example, in the context of mGluR-induced MMP9 hyperactivity (Bilousova et al., 2008). These results also indicate that targeting the misregulated activity-dependent mechanism of Dlp mediating activity-dependent overabundance of synaptic MMP1 could potentially prevent inappropriate connections caused by hyperexcitability in the FXS condition.

This activity-FMRP-HSPG-MMP regulatory mechanism of synapse remodeling presents questions. A key question is the means by which activity-dependent MMP1 localization is restored by reducing Dlp in the FXS model. It is probable that an unidentified synaptomatrix player is involved. Since Dlp can activate and inhibit Wg signaling (Yan et al., 2009; Dani et al., 2012), reducing Dlp could restore proportionality between interacting synaptomatrix components (**Figure 3**). Altered Dlp sulfation may change protease activity (Tocchi and Parks, 2013), perhaps in concert with other effectors, such as HSPG-sulfating *hs6st* and *sulf1* genes that modulate Wg signaling (Dani et al., 2012). Alternatively, Wg *trans*-synaptic signaling is reduced in the FXS condition (Friedman et al., 2013), and Wg itself may feedback to restore activity-dependent MMP1 function (**Figure 3**). Another possibility is that a synaptomatrix regulator preventing excess Dlp from misregulating activity-dependent MMP1 could be lost in the FXS condition. Indeed, FMRP can promote protein levels (Feng et al., 1997; Derlig et al., 2013), and this could include synaptomatrix proteins. For example, other HSPGs (e.g., Perlecan) might consolidate Wg signaling, thus restoring a more normal activity-dependent dynamic (**Figure 3**). Relevant MMP1 catalytic targets are unclear, though it is tempting to speculate secreted MMP1 may cleave Laminin-A to enable activity-dependent integrin signaling (Tsai et al., 2012; Lee et al., 2017). Whatever further elements of the synaptomatrix mechanism have yet to be elucidated, the finding that activity-dependent regulation can be restored in the *Drosophila* FXS disease model opens exciting possibilities for new FXS therapeutic treatments, and may lead to the discovery of novel activity-regulated extracellular molecules critical for synaptic remodeling.

## Future Directions

The very recent work discussed in this article highlights the utility of the *Drosophila* FXS disease model for the study of developmental activity-dependent mechanisms at synaptic connections, during use-dependent synaptic remodeling and in early-use critical periods. These new advances further demonstrate FMRP requirements in activity-dependent regulation of protein translation and control of signaling mechanisms operating at the heart of synapse formation and refinement. The particularly well-characterized central brain MB olfactory learning and memory circuitry has become a powerful vehicle for determining molecular mechanisms disrupted by FMRP loss, cellular mechanisms of activity-dependent synaptic remodeling, and the means of establishing excitatory/inhibitory synapse balance during the critical period (Doll et al., 2017; Vita and Broadie, 2017). In parallel, the classic GF visual escape circuit linking sensory input, brain integration and motor output via particularly large and well-characterized interneurons has the promise of providing an exciting new avenue to dissect FMRP requirements (Kennedy and Broadie, 2017). Finally, the malleable NMJ provides a large and genetically tractable glutamatergic synapse model, which continues to be instrumental in the discovery and elucidation of FMRP synaptic requirements, including intracellular signaling, intercellular interactions, and *trans*-synaptic pathways that strongly contribute to the FXS disease state (Dear et al., 2017). These diverse circuits will continue to be the focus of future studies, as we seek to determine generalizable FMRP requirements throughout the entire nervous system, as well as selective FMRP roles in specific neural circuits and synapses.

Our current understanding of FMRP requirements during the critical period paves the way for future studies examining molecular mechanisms of activity-dependent refinement. Based on recent findings (Doll and Broadie, 2016), we hypothesize that developmental misregulation of activity-induced Ca^2+^ signaling is a core contributor to the FXS condition. Importantly, classic memory-linked pathways (e.g., cAMP pathway) connect directly and indirectly to Ca^2+^ signaling (Davis and Dauwalder, 1991; Skoulakis et al., 1993; Kanellopoulos et al., 2012), with pathway members enriched in *Drosophila* brain MB and AL (**Figure 1**; Crittenden et al., 1998). FXS patient-derived cells and models similarly show reduced cAMP levels, and genetic/pharmacological correction of cAMP levels prevents FXS model phenotypes (Berry-Kravis and Huttenlocher, 1992; Berry-Kravis et al., 1995; Kelley et al., 2007; Kanellopoulos et al., 2012). Downstream of cAMP, PKA phosphorylates a wide range of neuronal targets (Sassone-Corsi, 2012), and enhances excitability in both excitatory and inhibitory neurons to promote activity-dependent remodeling (Lee, 2015). A likely downstream target, the small GTPase Rac1, acts as a molecular switch in structural and functional synaptic plasticity, and is of interest in the context of FXS hyperexcitability (Lee et al., 2003; Schenck et al., 2003; Bongmba et al., 2011; Goto et al., 2013, 2014; Tejada-Simon, 2015). Interestingly, inhibition of PAK downstream of Rac1 prevents FXS model phenotypes (Dolan et al., 2013). We therefore hypothesize that FXS phenotypes associated with aberrant Ca^2+^-cAMP-PKA-Rac1-PAK signaling likely occur in both the MB and AL during the early-use critical period (Doll et al., 2017; Vita and Broadie, 2017).

We are increasingly aware of possible intercellular interactions in the FXS state, such as neuron-glia roles in circuit refinement (Logan, 2017). Based on our recent work (Vita and Broadie, 2017), we hypothesize dysregulated neuronal surface signaling cues impair glia-mediated phagocytosis driving synaptic pruning during activity-dependent refinement (**Figure 2**). Specifically, we propose disrupted membrane trafficking due to elevated ESCRTIII Shrub levels could alter a surface signal for glial phagocytosis (Vita and Broadie, 2017). Consistently, glia-mediated developmental phagocytosis pruning of MB gamma neuron collateral branches is reduced/delayed in the absence of FMRP (O’Connor et al., 2017). Studies to date have focused primarily on glial clearance via the Draper/Ced-1/MEGF-10 receptor pathway (Musashe et al., 2016; Lu et al., 2017). We propose that FMRP loss may cause improper refinement through signaling defects that prevent glial phagocytosis, delay signaling processes that promote phagocytosis, or manifest aberrations in glial inability to sufficiently regulate or respond to other cells during critical period refinement. Interestingly, glia modulate the excitation/inhibition balance via a GABA uptake mechanism regulating synaptogenesis (Muthukumar et al., 2014). We therefore hypothesize that neuron-glia interactions may also modulate synaptic excitation/inhibition balance in critical period refinement. Recent work in mice shows glial FMRP is necessary, but not sufficient, for FXS model dendritic spine dynamics (Hodges et al., 2017), and co-cultures with astrocytes lacking FMRP illustrate delayed development (Jacobs et al., 2016), indicating that FMRP can act directly in glia as well as in neurons.

In the NMJ model, misregulation of Wnt Wg *trans*-synaptic signaling is an established cause of FXS phenotypes (Friedman et al., 2013). FMRP is required for activity-dependent HSPG Dlp regulation of extracellular MMP1 synaptic localization and enzymatic function (Dear et al., 2017). MMP1, in turn, is absolutely required for rapid synaptic bouton formation in response to activity. Moreover, Tissue Inhibitor of MMP (TIMP) overexpression prevents synaptic defects in the *Drosophila* FXS model (Siller and Broadie, 2011), suggesting that synaptomatrix protease regulation is another avenue worth investigating in the FMRP-Dlp-MMP1 pathway (Dear et al., 2017). HSPG Syndecan is negatively regulated by FMRP (Friedman et al., 2013), and may therefore also be involved. Downstream of altered Wnt Wg *trans*-synaptic signaling, defective Fz2-C nuclear import is well described in the *Drosophila* FXS disease model (Friedman et al., 2013), but it remains to be tested whether autocrine Wg signaling is also impacted. Based on work showing that inhibition of the Wg divergent canonical target GSK3β/Shaggy is a promising FXS therapeutic treatment (Klein and Melton, 1996; Stambolic et al., 1996; McBride et al., 2005; Choi et al., 2010; Mines and Jope, 2011), we hypothesize that overabundant synaptic Dlp sequesters Wg ligand, inhibiting Wg signaling, and therefore the activity-dependent suppression of GSK3β/Shaggy. Future work will test whether Wg sequestration by excess Dlp explains the activity-insensitivity of *dfmr1* null synapses. Synapse-associated glia also regulate Wg *trans*-synaptic signaling (Kerr et al., 2014; Kopke et al., 2017), indicating another plausible source of aberrant synaptomatrix regulation that needs to be explored in the FXS condition.

As we continue ongoing studies exploiting the *Drosophila* FXS disease model, we posit outstanding needs to dissect developmental activity-dependent synaptic remodeling and connectivity refinement mechanisms, both in the brain and at the NMJ. Within the brain AL-MB olfactory circuit (**Figure 1**), OSN, PN, and KC synaptic connections are well suited to pursue the mechanisms of *trans*-synaptic signaling (e.g., Notch, Wg), synaptomatrix regulation (e.g., HSPG, MMP), signal transduction (e.g., cAMP-PKA, actin cytoskeleton), and intercellular interactions (e.g., neuron-glia). This circuit is also ideal for testing mechanisms of excitation/inhibition balance (e.g., mPN2 vs. MBON-11; **Figure 1**) developing in response to sensory experience during early-use critical periods. Our work highlights a restricted, transient window of FMRP requirement coinciding with peak FMRP levels. In parallel, the NMJ glutamatergic model synapse will be instrumental for investigating the interplay of the multiple bidirectional *trans*-synaptic signaling pathways regulated by an increasingly defined synaptomatrix (**Figure 3**). This system is also ideal for testing activity-dependent synaptic remodeling mechanisms, including bouton addition and elimination, and glial involvement in the refinement of the pre- and post-synaptic sides of the synapse. In addition, NMJ findings will continue to inform and direct ongoing central brain studies. Our goal is to continue to discover cellular and molecular mechanisms of activity-dependent circuit remodeling that optimize behavioral performance, and to reveal the FMRP-dependent neurodevelopmental processes that go awry in FXS, so as to be able to devise effective new treatments for this devastating disease state.

## Author Contributions

JS and KB co-wrote this article.

## Conflict of Interest Statement

The authors declare that the research was conducted in the absence of any commercial or financial relationships that could be construed as a potential conflict of interest.
